# The Demographic and Clinical Characteristics of Ulcerative Colitis in a Northeast Brazilian Population

**DOI:** 10.1155/2015/359130

**Published:** 2015-10-05

**Authors:** Bruno César da Silva, Andre Castro Lyra, Carlos Maurício Cardeal Mendes, Camila Paula Oliveira Ribeiro, Sonyara Rauedys Oliveira Lisboa, Mariana Tinoco Lordello de Souza, Renata Cavalcanti Portela, Genoile Oliveira Santana

**Affiliations:** ^1^Postgraduate Program in Medicine and Health, Federal University of Bahia, Rua Augusto Viana s/n, 5 Andar, Canela, 40110-060 Salvador, BA, Brazil; ^2^Gastroenterology Department, Hospital da Bahia, Avenida Professor Magalhães Neto 1541, Pituba, 41820-011 Salvador, BA, Brazil; ^3^Gastroenterology and Hepatology Discipline, Department of Medicine, Federal University of Bahia, Rua Augusto Viana s/n, 5° Andar, Canela, 40110-060 Salvador, BA, Brazil; ^4^Institute of Health Sciences, Federal University of Bahia, Avenida Sete de Setembro, s/n, Canela, 40110-060 Salvador, BA, Brazil; ^5^Bahiana School of Medicine and Public Health, Rua Silveira Martins, 3386 - Cabula, 41150-100 Salvador, BA, Brazil

## Abstract

*Introduction*. The purpose of this study was to describe the clinical and demographic characteristics of UC in Bahia, a Brazilian state, and to identify the variables associated with extensive colitis, steroid therapy, immunosuppression, and colectomy. *Methods*. In this cross-sectional study UC patients were interviewed, and additional information was collected from the medical records. Descriptive statistics and multivariate Poisson regression analysis were used. *Results*. This study included 267 individuals, the mean age of whom was 39.4 years at diagnosis. There was a predominance of females and left-side colitis. Extensive colitis was positively associated with male gender, diarrhea, weight loss, and a younger age at diagnosis. In contrast, active smoking and a family history of IBD were negatively associated with extensive colitis. Positive associations were observed between steroid therapy and diarrhea, weight loss, urban patients, extraintestinal manifestations (EIMs), and hospitalization. Younger age and weight loss at diagnosis, a family history of IBD, extensive colitis, EIMs, hospitalization, and steroid therapy were all positively associated with immunosuppression. In contrast, Caucasian individuals, smokers, patients with rectal bleeding, and rural patients areas were all observed to have a decreased likelihood of immunosuppression. *Conclusions*. Our results corroborate the association between higher prevalence of extensive colitis and younger age at diagnosis. An association between steroid therapy and clinical presentation at diagnosis was observed. The observation that white individuals and rural patients use less immunosuppressive drugs highlights the need to study the influence of environmental and genetic factors on the behavior of UC in this population.

## 1. Introduction

In recent decades, the incidence of inflammatory bowel disease (IBD) has increased in several regions of the world, especially in developing countries [1, 2]. Although nationwide data on the incidence and prevalence of ulcerative colitis (UC) in Brazil are lacking, research results from several regions of the southeast have shown that the incidence of UC and the number of hospitalizations have been increasing over the past several years [3, 4].

Most publications that describe the demographic and clinical characteristics of patients with UC have been conducted in Europe or North America. However, even these studies from developed countries have been characterized by variable methods and usually have been conducted with specific populations within certain regions at medical reference centers [5]. Studies examining the demographic and clinical data of patients with UC are scarce in Latin America, including Brazil.

UC presents variable demographic and clinical characteristics in different regions of the world. Environmental and genetic factors appear to be related to this phenotypic heterogeneity [6]. For example, research has shown that the evolution of UC is usually less severe (i.e., there is a decreased need for surgery, a lower incidence of colorectal cancer, and fewer intestinal manifestations) in Asian countries than in developed Western countries [7, 8].

The Brazilian population is comprised of individuals from several different ethnic backgrounds, including those of European descents, Brazilian Indigenous descents, and African descents. Bahia is a state in which the population is composed primarily of individuals of African descent. This population results from Brazil's unique history since the sixteenth century. Knowing the characteristics of UC in the population of northeastern Brazil, especially in Bahia, is important because most prior publications regarding UC have been performed with predominantly Caucasian or Asian populations.

Our purpose was to describe the demographic and clinical characteristics of patients with UC from two IBD treatment referral centers in Bahia (a state in northeastern Brazil) and to identify the presence of factors associated with the extent of disease, the use of corticosteroids, the use of immunosuppressive therapy, and colectomy. Such studies are useful to evaluate the phenotypic differences in various regions of the world. In addition, this information may be able to guide research that could potentially elucidate the role of genetic and environmental factors in the etiology and natural history of the disease.

## 2. Methods

This cross-sectional study was performed between January 2011 and September 2012 with UC patients of the Professor Edgard Santos University Hospital and of the Roberto Santos General Hospital, the only two IBD treatment referral centers in Bahia, both of which are located in the city of Salvador. The patients were interviewed, and additional information was collected from medical records immediately following each interview. Individuals included in the study were diagnosed with UC based on clinical, endoscopic, radiological, and histological findings [9]. Patients with indeterminate colitis, Crohn's disease, or infectious and parasitic colitis were not included.

The following clinical and demographic characteristics were analyzed: gender, skin color, origin (urban or rural), the year when the diagnosis occurred, the age at the time of diagnosis, disease duration, the extent of UC according to the Montreal classification [10], symptoms (diarrhea, weight loss, abdominal pain, and blood in the stool), family history of IBD, smoking history, hospital admission, extraintestinal manifestations (EIMs), colectomy, and the use of corticosteroids, azathioprine, or antibodies against tumor necrosis factor (anti-TNF). For our multivariate regression models, the extent of disease was classified into two categories (i.e., with and without extensive colitis).

To classify patients according to skin color, the method adopted by the Brazilian Institute of Geography and Statistics (IBGE) was used [11], which allows individuals to declare themselves as black, pardo (mixed-race), white, yellow, or indigenous. In multivariate regression models, individuals identified as black, mixed-race, and Indian were grouped together to form the category of nonwhites.

In Bahia, the treatment of IBD is part of a special program of the government. For patients with UC, the first choice of aminosalicylate is sulfasalazine. If the patients progress with adverse events, it is recommended to use mesalazine. Patients with mild to moderate disease activity were treated with aminosalicylates (i.e., sulfasalazine—3.0 to 6.0 g/day; mesalazine—2.4 to 4.8 g/day; or suppository mesalazine—0.5 to 1.5 g/day). Individuals refractory to initial therapy were treated with systemic steroids orally (prednisone 40–60 mg/day), with a weaning regimen lasting approximately two months. Azathioprine was used for steroid dependent patients (2.0 to 2.5 mg/kg/day). Individuals with severe UC were hospitalized and treated with intravenous steroids (hydrocortisone 300–400 mg/day) [12]. Colectomy was indicated when there was no response to intravenous corticosteroid therapy after one week of treatment. Infliximab is not part of the UC treatment protocol adopted by the Brazilian government. However, in recent years, specific cases have been individually assessed by a technical chamber that has the prerogative to approve the use of infliximab.

Data were analyzed using the Statistical Package for the Social Sciences (version 21.0, SPSS, Chicago, IL) and the statistical package R (version 3.0.1, 2013). The population was characterized using descriptive statistics. Because the entire population of the two reference centers was examined (rather than a random sample) statistical inference was not performed [13]. The selection of variables to be included in each multivariate regression model to analyze the associations between variables accounted for the importance of each variable according to evidence from the literature and the authors' own experience. An association with a prevalence ratio equal to or greater than 20% was adopted as the criterion for keeping variables in the final model. Akaike information criterion (AIC) and residual analyses were used as criteria for the goodness of fit. Collinearity and overdispersed variance were also evaluated.

Written informed consent was obtained from each patient. The study was conducted in accordance with the Declaration of Helsinki and it was approved by the ethics committee of the University Hospital Professor Edgard Santos.

## 3. Results

**Figure 1:**
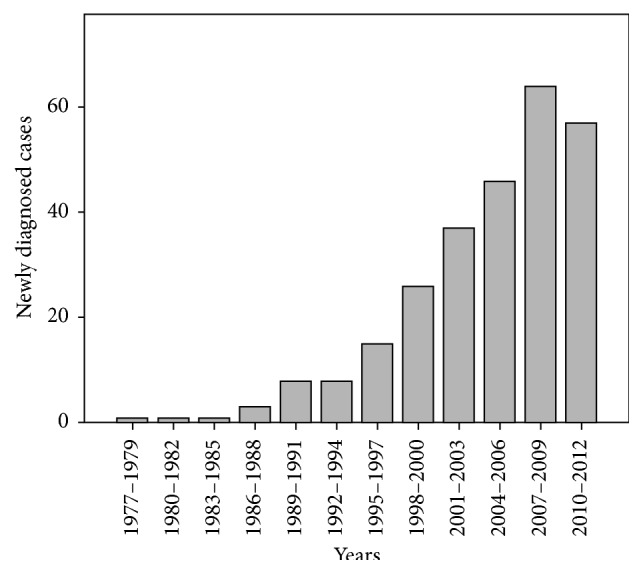
Distribution of patients according to the year of diagnosis of ulcerative colitis in two reference centers in Salvador, Bahia, Brazil (1977–2012).

A total of 267 patients were included in the study, of whom 180 (67.4%) were female and 218 (83.8%) were nonwhite. The majority of patients resided in urban areas, and the average age at the time of diagnosis was 39.4 years (with a range of 11–78 years). The mean duration of disease was 7.56 years (with a range of 0–34 years). The demographic characteristics of the patients are shown in Table 1.  Figure 1 shows the distribution of patients according to the year of diagnosis of ulcerative colitis in two reference centers in Salvador, Brazil.

Because colonoscopies were not performed, it was not possible to determine the extent of disease of 14 (5.2%) patients. There was a slight predominance of left colitis in the study population (42.7%). Among the symptoms presented at the time of diagnosis, the elimination of blood in the stool was the most frequent (74.8%). The clinical characteristics of the patients are shown in Table 2.

The Poisson regression model revealed a positive association between extensive colitis and the following factors: age at the time of diagnosis less than 30 years, male, diarrhea, and weight loss at the time of diagnosis. Individuals who had been diagnosed with UC prior to the age of 30 years had a prevalence of extensive colitis that was 58% higher than that observed in individuals diagnosed with UC when they were over 30 years of age. Male patients had a prevalence of extensive colitis that was 33% higher than females. The group that exhibited diarrhea at the time of diagnosis had a 67% increased prevalence of extensive colitis, and patients exhibiting weight loss had a prevalence of extensive colitis that was 51% higher than patients without this symptom. In contrast, active smoking and a family history of IBD were negatively associated with the occurrence of extensive colitis (Table 3).

In the Poisson regression model, systemic corticosteroid therapy was positively associated with the following variables: diarrhea and weight loss at the time of diagnosis, the occurrence of EIMs, and the need for hospitalization. Conversely, a smaller proportion of patients from rural areas used steroids (Table 4).

The factors associated with azathioprine usage were also evaluated using multivariate Poisson regression analysis. Being under 30 at the time of diagnosis, weight loss early in the disease, family history of IBD, extensive colitis, EIMs, previous hospitalization, and the use of corticosteroids were all shown to be positively associated with the use of azathioprine. In contrast, living in rural areas, active smoking, identifying as white, and rectal bleeding at the time of diagnosis were all shown to be protective factors against the use of immunosuppressive drugs (Table 5). On account of the numerical imbalance caused by the existence of only two cases of azathioprine usage in patients with diarrhea who had not previously used corticosteroids and due to a lack of individuals who used both azathioprine and corticosteroids but did not have diarrhea, diarrhea was not included as a variable in the Poisson regression model.

Due to the small number of patients treated surgically, it was not possible to perform a multivariate regression analysis to assess the factors associated with colectomy.

## 4. Discussion

This is the first study to evaluate clinical and demographic characteristics in patients with UC in Bahia, a state in northeastern Brazil. In general, the phenotypic characteristics of UC observed in our population are similar to those of other published studies. Our results help to reinforce concepts already well established, such as the association between extensive colitis and the diagnosis of UC at younger age and the protection conferred by smoking against more severe forms of UC. Because of the importance that is currently attributed to the influence of environment and genetics on the pathogenesis of IBD and the relationships of these factors with the natural course of the disease, we emphasize that our results reveal a lower prevalence of the use of steroids and immunosuppressants in patients from rural areas and a lower use of immunosuppressants by the white population. These results encourage us to attempt to discover the precise genetic and environmental factors that impact UC in these populations.

Although some studies, especially those conducted in high incidence areas of IBD, have reported a higher prevalence of IBD in men or an equal distribution between the genders [14–17], approximately two-thirds of our patients were female. Similar results have also been observed in studies of Brazilian and Portuguese patients [4, 18].

Our study population was primarily composed of nonwhites. It was observed that the prevalence of azathioprine usage in white patients was 44% lower than in nonwhites. This result suggests that nonwhite patients require more intensive therapy over time, at least in our environment. In contrast, a systematic review by Mahid and colleagues revealed no significant differences in the phenotypic presentation and severity of IBD between groups of white and black Americans [19].

The percentage of UC patients with a family history of IBD is variable and appears to be higher in Caucasians and in countries with the highest incidence of UC. In a study of the US population, 14.6% of UC patients had a family history of IBD [14]. In contrast, this rate is less than 2% in eastern populations [20]. Compared to these studies, our study identified an intermediate percentage of UC patients with a family history of IBD (7.9%), which is similar to the results described by Portela et al. for the Portuguese population (7.2%) [21]. The lower occurrence of family history and increased incidence of UC in less developed countries in recent decades suggests that the populations in these countries may have increased exposure to environmental triggers of IBD. Our results indicate that the use of immunosuppression is 26% higher in patients with a family history of IBD. In a prospective study, Henriksen et al. found no significant differences in drug therapy or indications for colectomy in the groups with or without a family history of IBD [22].

Extensive colitis and a greater need for azathioprine were both more common in individuals who were less than 30 years old at the time of disease diagnosis. In an Italian study of 1705 UC patients, younger patients were observed to present with more severe disease evolution and a greater need for more intensive treatment [23]. The IBSEN study showed that the risk of colectomy was higher in individuals diagnosed with UC before the age of 50 [24]. Despite these publications, there is no consensus regarding the influence of age at the time of diagnosis in the natural history of UC.

The extent of UC is a factor that influences disease progression and the long-term prognosis for patients [14, 25]. Solberg et al. followed patients with UC for 10 years and noted that individuals with extensive colitis showed an increased need for colectomy [24]. Barreiro-de Acosta et al. demonstrated that extensive colitis is a factor associated with an increased need for immunosuppression and biological therapy [18]. In our study, the use of azathioprine was 41% higher in patients with extensive colitis.

In our study, the occurrence of weight loss, diarrhea, and EIMs at the time of diagnosis and a greater need for hospitalization were variables significantly associated with the use of corticosteroids. However, there have been no publications with the main objective of describing the factors that are associated with the use of corticosteroids in UC patients.

The use of azathioprine in UC is indicated in patients who are steroid dependent and in patients who are refractory to aminosalicylates [12]. The identification of factors associated with the use of thiopurines has not been extensively explored, but it has the potential to help predict the patients who are likely to develop severe UC and thus require more intensive therapy. In our study, a positive association was observed between the use of azathioprine and several other factors, including receiving an initial UC diagnosis when under the age of 30, weight loss at the time of diagnosis, EIMs, a family history of IBD, extensive colitis, hospitalization, and a history of systemic corticosteroid therapy. In contrast, living in rural areas was negatively associated with azathioprine use. In our state, patients living in rural and urban areas have similar access to thiopurines through a government program. A Canadian study published in 2008 also revealed an association between the use of azathioprine and the extent of UC [26].

Unlike Crohn's disease, smoking has been recognized as a protective factor against the development of UC [27]. Moreover, among patients with UC, those who do not smoke exhibit a worse disease prognosis [28]. Our results demonstrate that active smoking is associated with a lower prevalence of extensive colitis (PR: 0.30) and less use of immunosuppression (PR: 0.75) in our study population.

Individuals residing in urban areas have a higher risk of developing UC compared to those living in rural areas [29]. In our study, 87.6% of the study sample lived in urban areas and only 12.4% lived in rural areas. In contrast, according to IBGE data, approximately 28% of the population of Bahia lives in rural areas [30]. It is possible that this lower percentage of UC patients from rural areas compared to the total percentage of individuals from rural areas in the general population may result from decreased exposure to environmental factors involved in the pathophysiology of IBD. Furthermore, our results showed that living in a rural area was associated with a decreased need for corticosteroids and immunosuppression. Consistent with this, a study by Ananthakrishnan et al. concluded that UC patients from areas with high levels of pollutant emissions have an increased risk of hospitalization (RR 1.48; 95% CI: 1.27–1.73) [31].

In our study, the presence of EIMs was associated with an increased use of steroids (PR: 1.32) and azathioprine (PR: 1.25). At least one previous study concluded that the presence of EIMs is associated with a greater extent of disease [32]. In addition, a recent study described an association between the presence of EIMs and an increased rate of colectomy in the pediatric population [33].

The colectomy rates reported in published studies have been highly variable, mainly due to the year and the geographical area in which they were performed. In Western countries, colectomy rate has been reduced over time. Recent studies indicate a colectomy rate around 10% [24, 34, 35]. The increased use of immunosuppressants and anti-TNF has been implicated as responsible for this decline [36]. The colectomy rate found in our population was 3.4%. In a retrospective study, a similar result was described by Park et al., who reported cumulative colectomy rates of 2%, 2.8%, and 3.3% at 1, 3, and 5–15 years, respectively [37]. In our study, environmental factors and a higher rate of using immunosuppressive drugs may have contributed to this low colectomy rate. However, because this is a cross-sectional study, it is possible that this rate may be underestimated due to the loss of follow-up of patients who may have eventually undergone colectomies.

In conclusion, the occurrence of UC in patients who were receiving specialized IBD treatment at centers in the state of Bahia increased over time. There was a predominance of females and left-side colitis in the present sample, but in general, the clinical characteristics of UC patients in Bahia are similar to those reported in the literature. Our results also corroborate the higher prevalence of extensive colitis in patients who were diagnosed at a younger age. Association between steroid therapy and clinical presentation at diagnosis was observed. Several of our results, including the negative association between living in rural areas and the use of azathioprine or systemic corticosteroids and the reduced incidence of azathioprine use by the white subgroup, highlight the need for additional studies examining the effects of environmental and genetic factors on the progression of UC in our study population.

## Figures and Tables

**Table 1:** Demographic characteristics of patients with ulcerative colitis in two reference centers in Salvador, Bahia, Brazil (*n* = 267).

Variables	*n* (%)
Gender	
Male	87 (32.6)
Female	180 (67.4)
Skin color	
Blacks	69 (26.5)
Mixed-race	147 (56.5)
Whites	42 (16.2)
Yellows	—
Indigenous	2 (0.8)
Origin	
Urban areas	234 (87.6)
Rural areas	33 (12.4)
Age at diagnosis (in years)	
Average	39.4; SD = 13.5
Range	11–78
Interval between symptom onset and diagnosis (in months)	
Average	20.36; SD = 31.3
Range	0–180
Mean disease duration (in years)	
Average	7.56; SD = 6.3
Range	0–34
Smoking	
Active	8 (3.0)
Former	92 (34.5)
No	167 (62.5)
Family history of IBD	
Yes	21 (7.9)
No	245 (92.1)

SD = standard deviation. IBD = inflammatory bowel disease.

**Table 2:** Clinical characteristics of patients with ulcerative colitis in two reference centers in Salvador, Bahia, Brazil (*n* = 267).

Variables	*n* (%)
Extension according to the Montreal classification	
Proctitis	41 (16.2)
Left colitis	108 (42.7)
Extensive colitis	104 (41.1)
Predominant symptoms at diagnosis	
Blood in stool	199 (74.8)
Diarrhea	143 (53.8)
Abdominal pain	97 (36.5)
Weight loss	26 (9.8)
Extraintestinal manifestations	
Joint	93 (34.8)
Cutaneous	9 (3.4)
Hepatobiliary	6 (2.2)
Eye	2 (0.7)
Need for hospitalization	
Yes	117 (43.8)
No	150 (56.2)
Use of azathioprine	
Yes	52 (19.5)
No	215 (80.5)
Use of corticosteroids	
Yes	167 (62.8)
No	99 (37.2)
Biological therapy	
Yes	4 (1.5)
No	263 (98.5)
Colectomy	
Yes	9 (3.4)
No	258 (96.6)

**Table 3:** Poisson regression model of the factors associated with extensive colitis in ulcerative colitis patients in two reference centers in Salvador, Brazil.

Variables	Initial model		Final model	
	PRadj^*∗∗*^	Coefficent^*∗*^	PRadj^*∗∗*^	Coefficent^*∗*^
(Intercept)		−1.17		−1.39
Age (years)		0.45		0.45
<30	1.56		1.58	
≥30	1		1	
Gender		0.29		0.28
Male	1.34		1.33	
Female	1		1	
Family history of IBD		−0.33		−0.33
Yes	0.71		0.71	
No	1		1	
Weight loss		0.49		0.51
Yes	1.64		1.67	
No	1		1	
Diarrhea		0.38		0.41
Yes	1.46		1.51	
No	1		1	
Active smoking		−1.20		−1.17
Yes	0.29		0.30	
No	1		1	
Origin		−0.03		—
Rural areas	0.96		—	
Urban areas	1		—	
White		−0.04		—
Yes	0.95		—	
No	1		—	
Blood in stools		−0.07		—
Yes	0.93		—	
No	1		—	
EIMs		−0.01		—
Yes	0.99		—	
No	1		—	

Note: Akaike information criterion (AIC): 383.

^*∗*^Coefficent from Poisson regression model.

^*∗∗*^Adjusted prevalence ratio from the Poisson regression model.

EIMs = extraintestinal manifestations.

IBD = inflammatory bowel disease.

**Table 4:** Poisson regression model of the factors associated with systemic corticosteroid therapy in patients with UC in two reference centers in Salvador, Brazil.

Variables	Initial model		Final model	
	PRadj^*∗∗*^	Coefficent^*∗*^	PRadj^*∗∗*^	Coefficent^*∗*^
(Intercept)				–0.98
Weight loss		0.26		0.25
Yes	1.30		1.29	
No	1		1	
Diarrhea		0.20		0.24
Yes	1.23		1.27	
No	1		1	
EIMs		0.29		0.28
Yes	1.34		1.32	
No	1		1	
Origin		–0.28		–0.29
Rural areas	0.75		0.74	
Urban areas	1		1	
Need for hospitalization		0.57		0.51
Yes	1.78		1.67	
No	1		1	
Age (years)		0.11		—
<30	1.12		—	
≥30	1		—	
Gender		–0.19		—
Male	0.82		—	
Female	1		—	
Blood in stool		0.03		—
Yes	1.04		—	
No	1		—	
Active smoking		0.18		—
Yes	1.21		—	
No	1		—	
Family history of IBD		0.002		—
Yes	1.0		—	
No	1		—	
White		0.06		—
Yes	1.06		—	
No	1		—	
Extensive colitis		0.16		—
Yes	1.18		—	
No	1		—	

Note: Akaike information criterion (AIC): 479.2.

^*∗*^Coefficent from Poisson regression model.

^*∗∗*^Adjusted prevalence ratio from the Poisson regression model.

EIMs = extraintestinal manifestations.

IBD = inflammatory bowel disease.

**Table 5:** Poisson regression model of the factors associated with the use of azathioprine in patients with UC in two reference centers in Salvador, Brazil.

Variables	Initial model		Final model	
	PRadj^*∗∗*^	Coefficient^*∗*^	PRadj^*∗∗*^	Coefficient^*∗*^
(Intercept)		−3.34		−3.92
Age (years)		0.54		0.55
<30	1.72		1.73	
≥30	1		1	
Origin		−0.69		−0.66
Rural areas	0.50		0.51	
Urban areas	1		1	
Family history of IBD		0.24		0.23
Yes	1.27		1.26	
No	1		1	
White		−0.58		−0.56
Yes	0.55		0.56	
No	1		1	
Active smoking		−0.26		−0.28
Yes	0.76		0.75	
No	1		1	
Blood in stool		−0.32		−0.33
Yes	0.72		0.71	
No	1		1	
Weight loss		0.37		0.37
Yes	1.45		1.45	
No	1		1	
EIM		0.24		0.22
Yes	1.27		1.25	
No	1		1	
Extensive colitis		0.33		0.34
Yes	1.40		1.41	
No	1		1	
Need for hospitalization		0.53		0.56
Yes	1.71		1.75	
No	1		1	
Use of corticosteroids		2.09		2.07
Yes	8.10		7.96	
No	1		1	
Gender		0.12		—
Male	1.13		—	
Female	1		—	
Diarrhea^†^		—		—
Yes	1.37		—	
No	1		—	

Note: Akaike information criterion (AIC): 221.6.

^†^Gross prevalence ratio.

^*∗*^Coefficient from the Poisson regression model.

^*∗∗*^Adjusted prevalence ratio from the Poisson regression model.

EIM = extraintestinal manifestations.

IBD = inflammatory bowel disease.
